# Metal Complex as a Novel Approach to Enhance the Amorphous Phase and Improve the EDLC Performance of Plasticized Proton Conducting Chitosan-Based Polymer Electrolyte

**DOI:** 10.3390/membranes10060132

**Published:** 2020-06-25

**Authors:** Ahmad S. F. M. Asnawi, Shujahadeen B. Aziz, Muaffaq M. Nofal, Yuhanees M. Yusof, Iver Brevik, Muhamad H. Hamsan, Mohamad A. Brza, Rebar T. Abdulwahid, Mohd F. Z. Kadir

**Affiliations:** 1 Chemical Engineering Section, Universiti Kuala Lumpur, Malaysian Institute of Chemical & Bioengineering Technology (UniKL MICET), Alor Gajah 78000, Malacca, Malaysia; asyafiq.asnawi@s.unikl.edu.my; 2 Hameed Majid Advanced Polymeric Materials Research Lab., Department of Physics, College of Science, University of Sulaimani, Qlyasan Street, Sulaimani 46001, Kurdistan Regional Government, Iraq; mohamad.brza@gmail.com (M.A.B.); rebar.abdulwahid@univsul.edu.iq (R.T.A.); 3 Department of Civil Engineering, College of Engineering, Komar University of Science and Technology, Sulaimani 46001, Kurdistan Regional Government, Iraq; 4 Department of Mathematics and General Sciences, Prince Sultan University, P.O. Box 66833, Riyadh 11586, Saudi Arabia; muaffaqnofal@gmail.com; 5 Malaysian Institute of Chemical and Bio-Engineering Technology, Universiti Kuala Lumpur (UniKL MICET), Alor Gajah 78000, Malacca, Malaysia; yuhanees@unikl.edu.my; 6 Department of Energy and Process Engineering, Norwegian University of Science and Technology, N-7491 Trondheim, Norway; iver.h.brevik@ntnu.no; 7 Institute for Advanced Studies, University of Malaya, Kuala Lumpur 50603, Gombak, Malaysia; hafizhamsan93@gmail.com; 8 Department of Manufacturing and Materials Engineering, Faculty of Engineering, International Islamic University of Malaysia, Kuala Lumpur 50603, Gombak, Malaysia; 9 Centre for Foundation Studies in Science, University of Malaya, Kuala Lumpur 50603, Malaysia; mfzkadir@um.edu.my

**Keywords:** plasticized polymer electrolyte, metal complex, XRD study, impedance study, EDLC fabrication

## Abstract

This work indicates that glycerolized chitosan-NH_4_F polymer electrolytes incorporated with zinc metal complexes are crucial for EDLC application. The ionic conductivity of the plasticized system was improved drastically from 9.52 × 10^−4^ S/cm to 1.71 × 10^−3^ S/cm with the addition of a zinc metal complex. The XRD results demonstrated that the amorphous phase was enhanced for the system containing the zinc metal complex. The transference number of ions (*t_ion_*) and electrons (*t_e_*) were measured for two of the highest conducting electrolyte systems. It confirmed that the ions were the dominant charge carriers in both systems as *t_ion_* values for CSNHG4 and CSNHG5 electrolytes were 0.976 and 0.966, respectively. From the examination of LSV, zinc improved the electrolyte electrochemical stability to 2.25 V. The achieved specific capacitance from the CV plot reveals the role of the metal complex on storage properties. The charge–discharge profile was obtained for the system incorporated with the metal complex. The obtained specific capacitance ranged from 69.7 to 77.6 F/g. The energy and power densities became stable from 7.8 to 8.5 Wh/kg and 1041.7 to 248.2 W/kg, respectively, as the EDLC finalized the cycles.

## 1. Introduction

Biopolymers are naturally abundant, low in cost, have high compatibility with solvents and are very stable in forming a film [1,2]. Researchers commonly use biopolymers like cellulose, starch and carrageenan as polymer hosts in polymer electrolytes [3,4,5]. Chitosan is also one of the biopolymers that is extensively studied in energy storage devices, as well as environmental and biomedical approaches [6]. Based on the chitosan chemical structure, there are different types of oxygen functional groups that are enriched in electron lone pairs. The conduction mechanism of an electrolyte is affected by the ions from the incorporated salt that has the ability to create a dative bond with these functional groups [7]. Liquid electrolytes are widely used because of their high performance in various energy devices. However, they are easy to evaporate and damage equipment caused due to leaking and corrosion [8,9]. Therefore, solid polymer electrolytes (SPEs) have been introduced because of their advantages compared to liquid electrolytes, namely that they are safe, allow for easy fabrication and have a long shelf life [10]. Biopolymer SPEs can reduce plastic waste pollution since they are made of natural resources and are highly biodegradable.

On the other hand, electrochemical double-layer capacitor (EDLC) is an alternative for conventional batteries used nowadays. The energy storage mechanism of EDLC is based on the non-faradaic process where the ions make a double layer at the interfacial region [11]. This means charge accumulation only occurs between the electrode surfaces but there is no electron transfer. Several materials have been used to fabricate the electrode for EDLC such as graphite [12], carbon aerogel [13], carbon nanotubes [14] and activated carbon [15]. Activated carbon has been widely explored in the electrolyte field due to its large surface area, good chemical durability and high electronic conductivity [16]. EDLC is more preferable than other supercapacitors because it has high power density, high durability and better thermal stability as well as a low fabrication cost with a straightforward methodology of EDLC devices [17,18,19]. Moreover, zinc-based electrochemical devices may provide equally good performance in the context of safety at ambient conditions because zinc is basically nontoxic, non-explosive, and inexpensive [20]. The availability of zinc is also found to be more abundant than lithium (Li). Furthermore, the sizes of Li^+^ and Zn^2+^ ions are quite comparable [21].

SPEs that have high conductivity (approximately 10^−4^ to 10^−3^ S/cm) are important for good performance of electrochemical storage devices. A few methods have been introduced to improve the conductivity of an electrolyte such as salt impregnation and plasticization [22,23]. Ammonium salts are a common ionic source in polymer electrolyte preparation. This is because these salts are able to replace other ionic sources such as inorganic salts to avoid the use of expensive and harmful lithium metal electrodes in lithium battery applications [24]. Ammonium salts have also been reported to be a good H^+^ provider to the polymer electrolytes due to their compatibility, high ionic conductivity as well as thermal stability [25]. Based on our previous work, there is an increment of conductivity for pure dextran film from (2.00 ± 0.70) × 10^−9^ S/cm to (2.23 ± 0.76) × 10^−3^ S/cm with the addition of 40 wt.% ammonium fluoride (NH_4_F) [26]. It has also been reported that the addition of NH_4_F salt into carboxymethylcellulose (CMC) film has increased the ionic conductivity of the electrolyte [27]. The dissociation of salt is affected by the plasticization process that provides more conducting paths for the mobile ions to migrate, thus increasing the ionic conductivity [28]. Meanwhile, Chai and Isa [29] mentioned that glycerol has the ability to increase the ionic mobility of the CMC–oleic acid electrolyte system and hence enhance ionic conductivity. According to Kumar et al. [30], maximum conductivity of 1.26 × 10^−4^ S/cm is achieved for glycerolized polyvinylidene fluoride (PVDF)–NH_4_F polymer electrolyte. A comparable conductivity is also obtained by glycerolized chitosan–NH_4_F polymer electrolyte [31]. Thus, glycerol has been chosen in this present work to be added into chitosan-based polymer electrolytes with an H^+^ ion to further enhance the conductivity. In our previous work, it was shown that metal complexes are crucial in improving the amorphous phase of polar polymers [32]. The increase of the amorphous phase is promising to increase DC ionic conductivity. Thus, the main objective of increasing the zinc metal complex into the chitosan-based electrolyte is to improve an amorphous phase for ion conduction. The highest conducting plasticized system among the electrolytes with and without the metal complex is employed for EDLC fabrication. The results shown in the present work indicate that the metal complex can enhance the performance of the fabricated EDLC device. The XRD and impedance investigations explore more evidence.

## 2. Materials and Sample Preparation

### 2.1. Materials

In the present work, chitosan with a relatively high molecular mass of around 310,000 to 375,000 g/mol, ammonium fluoride (NH_4_F) salt and glycerol in the fabricating of plasticized systems was used. All chemicals were purchased from Sigma-Aldrich (Missouri, MO, USA) without further purification.

### 2.2. Polymer Electrolyte Preparation

The procedure consists of 1 g insertion of CS into 50 mL acetic acid (1%) solution. Next, 40 wt.% in ammonium fluoride (NH_4_F) salt with a fixed amount was inserted to the provided solution above. The solution was stirred constantly with a magnetic stirrer at surrounding temperature until a homogenous solution developed. Then, glycerol with different quantities was added to the provided solution of polymer and salt together, with constant stirring until a clear solution emerged. The glycerol amount in the fabricated electrolytes was subjected to change from 0 to 40 wt.% in 10 wt.% steps. The plasticized samples were named as CSNHG0, CSNHG1, CSNHG2, CSNHG3 and CSNHG4. In addition, the plasticized sample of CS:NH_4_F electrolyte with 40 wt.% glycerol was incorporated with 10 mL of diluted zinc metal complex and named as CSNHG5. The methodology of the preparation of the zinc metal complex using green approaches can be seen in our previous work [32]. Finally, the mixed solutions were spilled into Petri dishes and enclosed with filter paper to avoid any pollution. The Petri dishes were left to evaporate solvent gradually at the surrounding temperature and to fabricate dry as well as a free-standing plasticized system.

### 2.3. EIS Analysis

Electrical impedance spectroscopy (EIS) was employed to measure the impedance of an electrolyte in this work by using HIOKI 3532-50 LCR HiTESTER (50 Hz ≤ *f* ≤ 5000 kHz) at room temperature. The chosen electrolyte was placed between two stainless steel discs before measurements took place. The data collected from this analysis was used to study the ionic conductivity.

### 2.4. Electrolyte Characterization

#### TNM and LSV Measurements

A digital DC power supply, V&A Instrument (Neware, Shanghai, China) DP3003 was used to conduct the transference number (TNM) analysis by using the DC polarization technique. Two blocking stainless steel electrodes in a Teflon holder were used to sandwich the highest conducting electrolyte. The electrode polarization was carried out at a constant potential of 0.8 V and the DC current was recorded as a function of time at room temperature. The potential window of the highest conducting electrolyte was also measured by using linear sweep voltammetry (LSV) analysis (DY2300 potentiostat, Neware, Shanghai, China) at a scan rate of 10 mV/s.

### 2.5. EDLC Preparation

The electrode for EDLC fabrication was made of polyvinylidene fluoride (PVdF), activated carbon and carbon black materials. Our previous work reported the detail of electrode preparation [33]. The thickness of the electrodes was optimized at 25 µm. Then, the highest conducting electrolyte was placed between two activated carbon electrodes and packed in CR2032 coin cells. The cyclic voltammetry (CV) of an EDLC system was conducted using Digi-IVY DY2300 Potentiostat in a voltage range between 0.0 to 0.9 V and at different sweep rates (10 to 100 mV/s). Specific capacitance (*C_s_*) can be calculated from the CV curve by using the following equation [34]:

(1) $$C_{s}=\int_{V_{i}}^{V_{f}}\frac{I\left(V\right)dV}{2m\upsilon \left(V_{2}-V_{1}\right)},$$

where *I(V)dV* is the area of the CV curve, while *m* and *v* are the mass of active material and scan rate, respectively. *V*_2_ and *V*_1_ in this work are 0.9 and 0.0 V, respectively. Charge–discharge profiles of the EDLC were tested using Neware battery cycler with the current density of 0.5 mA/cm^2^. *C_s_* value from charge–discharge profiles and equivalent series resistance (ESR) were calculated using the equations below [35,36]:

(2) $$C_{s}=\frac{i}{ms};$$

(3) $$ESR=\frac{V_{drop}}{i},$$

where *i* is the applied current, *s* is the slope of discharge part, and *V_drop_* is the voltage drop.

Equation (1), was used to calculate the *C_s_* values from the CV plots, while Equation (2), was used to determine the *C_s_* values from the discharge part of the charge–discharge profile. The value of the *C_s_* from Equation (1) varied with various scan rates of 10, 20, 50 and 100, while the value of the *C_s_* from the charge–discharge profiles was obtained for an applied current of 1 mA. The *C_s_* values from the CV curves were compared with the values of *C_s_* that were obtained from the charge–discharge curves. In addition, the *V_drop_* was measured from the charg–discharge curves and used to determine the ESR values using Equation (3).

## 3. Results and Discussion

### 3.1. XRD Analysis

**Figure 1:**
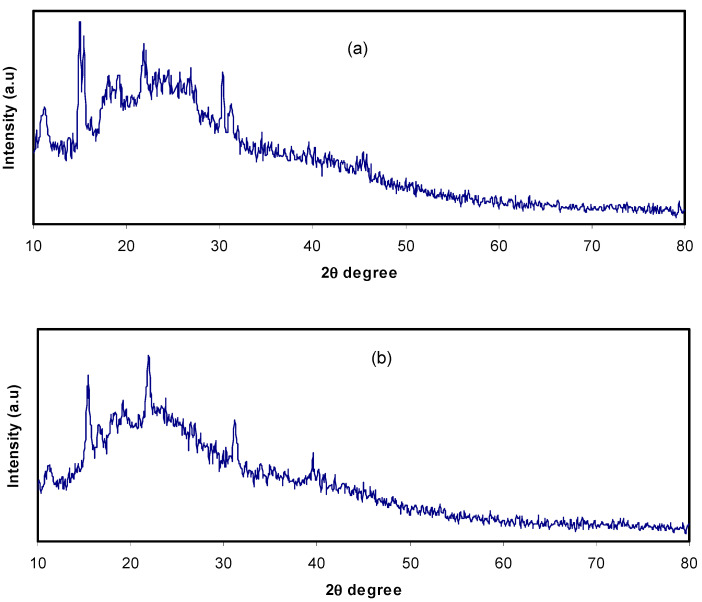

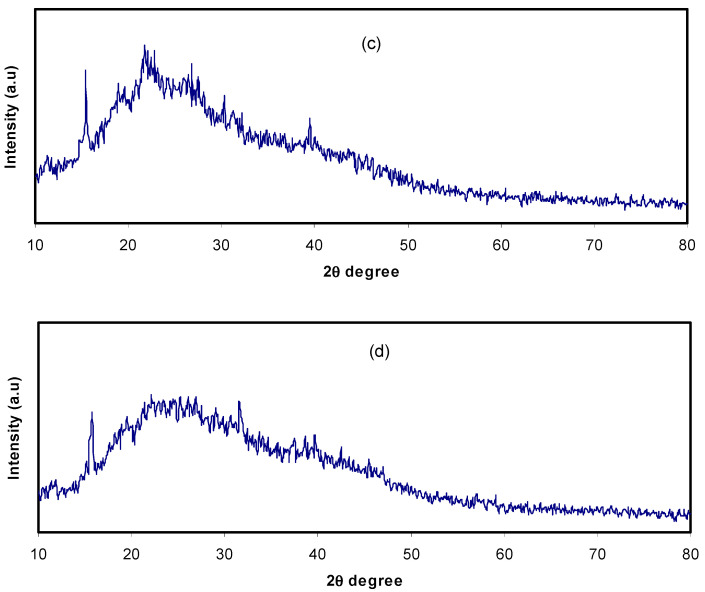
XRD patterns for (**a**) CSNHG0, (**b**) CSNHG2, (**c**) CSNHG4 and (**d**) CSNHG5.

Today, plasticized polymer electrolytes have been widely applied in electrochemical devices, for example smart windows, fuel cells and secondary batteries. The plasticizer inclusion into polymer electrolytes can enhance the electrical and electrochemical characteristics due to modification in structure [37]. The spectrum of XRD for CS:NH_4_F electrolyte films isdemonstrated in Figure 1a. It was clearly detected that the plasticized system was nearly amorphous in structure with a small number of crystalline peaks which ascribed to the complex creation between polymer and salt rather than the pure salt of NH_4_F. It can be seen that with an increase inthe amount of glycerol from 20 wt.% to 40 wt.%, the peaks extended and intensity reduction happened, as demonstrated in Figure 1b,c. In addition, the provided crystalline peaks from the complexes between the polymer and salt declined in the plasticized electrolyte systems. Previous reports have indicated that polymers with small molecular weight or comprised of nonvolatile organic solvents are well-known plasticizers. Herein, numerous plasticizer examples have been offered, for instance ethylene carbonate (EC), polyethylene glycol (PEG-200, PEG-400, PEG-600), propylene carbonate (PC) and dimethyl form amide (DMF) [38,39]. The properties of these fillers are due to their large dielectric constant which aids to expand the structure of amorphous in host polymers and assists the increment in degree of dissociation of inorganic salts, thus improving conductivity [39]. The high value (42.5) of the glycerol dielectric constant (ε), reduces the force of attraction among the cations and anions of the salts and among the chains of the polymers [23]. From astructural viewpoint, glycerol (C_3_H_8_O_3_) hasthree hydroxyl groups (–OH), demonstrating the obtainability of extra lone pairs of electrons for ionic conduction.

Due to plasticizer insertion, the increase in polymer chains’ flexibility consequently improved the conductivity of ions. The system incorporated with 10 mL of the Znmetal complex was more amorphous compared to those containing only glycerol (Figure 1d). These results indicate that metalcomplexes provide more amorphous phases and consequently more pathways would be available for ion conduction. In our previous work [32], it was established that metal complexes interact with the functional groups in the polymer and disrupt hydrogen bonding among polymer chains.

### 3.2. Impedance Study

**Figure 2:**
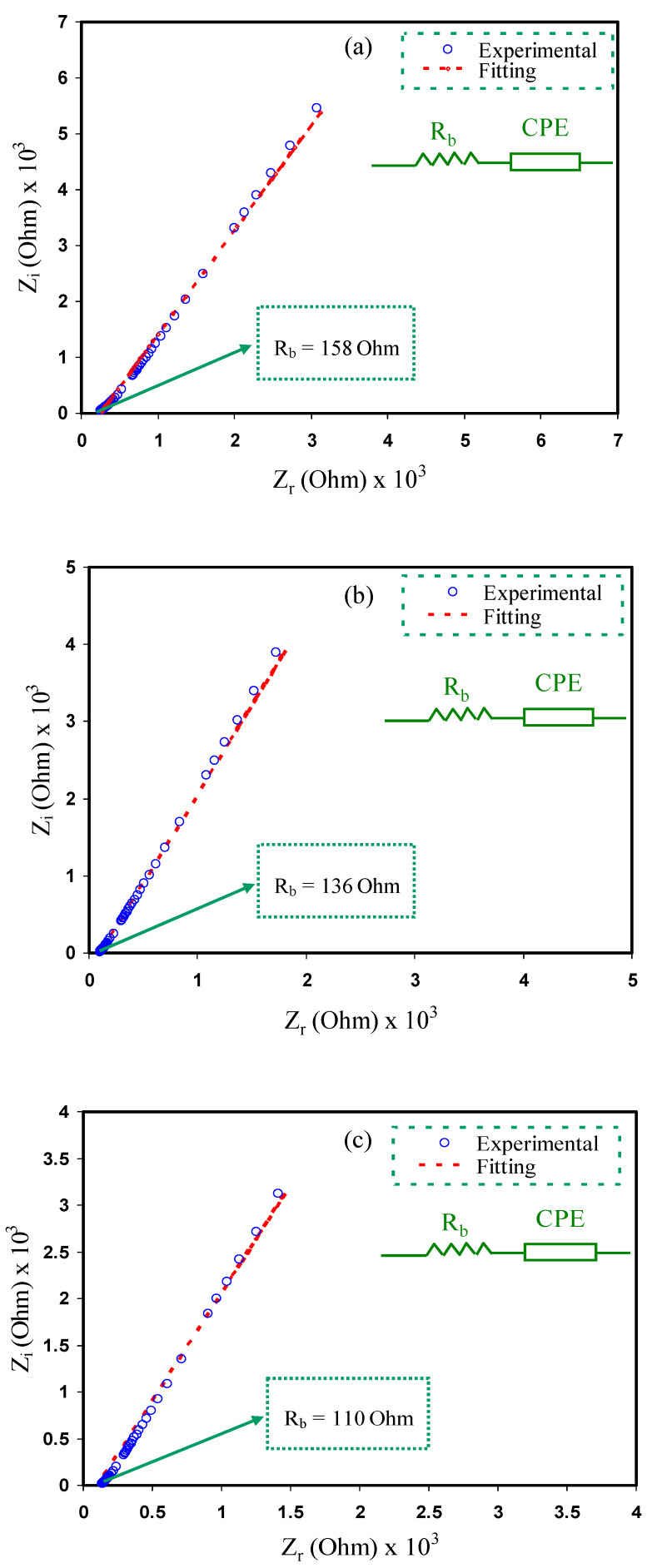

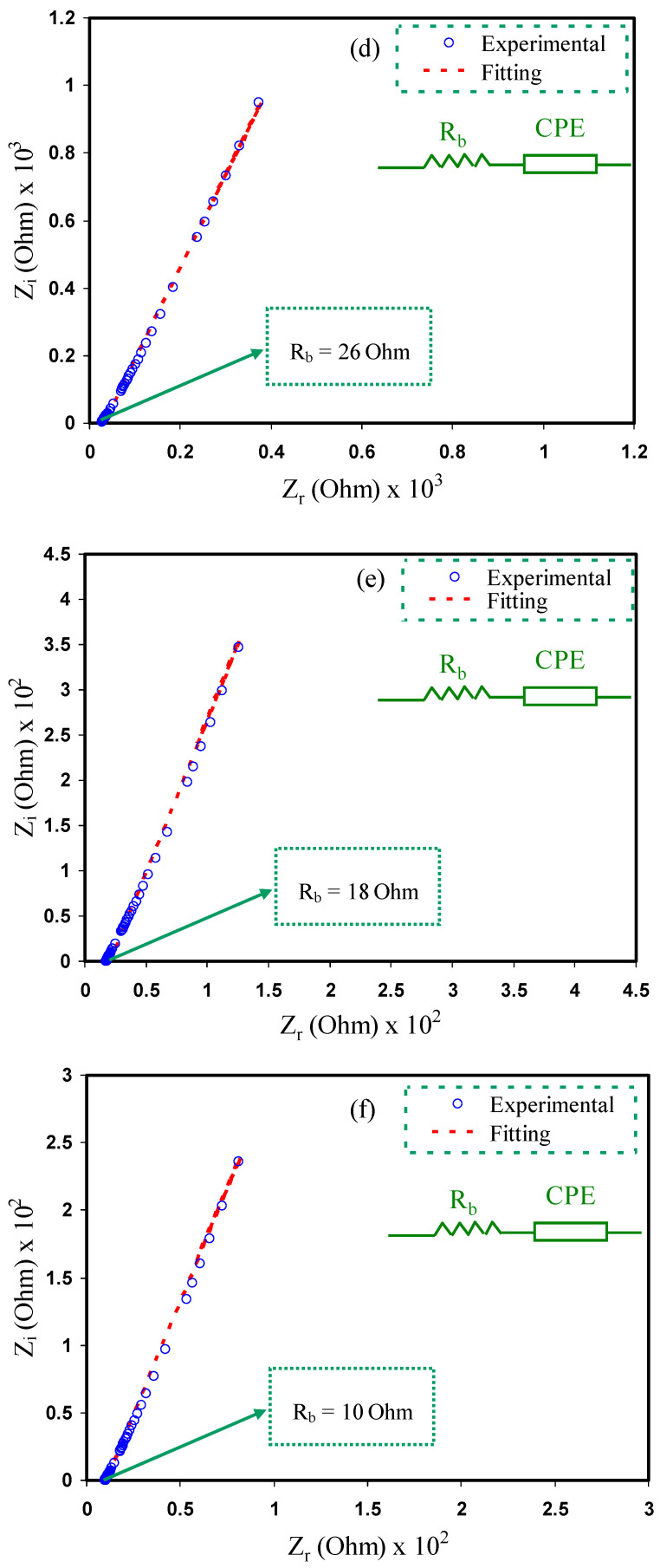
Impedance plots for (**a**) CSNHG0, (**b**) CSNHG1, (**c**) CSNHG2, (**d**) CSNHG3, (**e**) CSNHG4 and (**f**) CSNHG5.

Impedance spectroscopy is an efficient method to examine the ionic conductivity of polymeric materials and is also used to analyze the electrical properties of new materials that will be applied in electrochemical devices [40,41]. Due to the wide range of applicationsof solid electrochemical devices, the attention of ion conducting materials has been focused over the decade [42]. The typical results for impedance studies of polymer electrolytes consists of a semicircle and a spike at high and low frequency regions, respectively [43]. The impedance plots of CS:NH_4_F:Gly electrolyte films at room temperature are shown in Figure 2a–f.

The electrical equivalent circuit (EEC) technique was performed to study the electrochemical impedance spectroscopy (EIS) since this method is effortless, quick and produces a complete picture of the electrolyte system [38]. The Nyquist plots for the polymer electrolyte systems weregained in respect of the equivalent circuit (EC), which contains *R_b_* for the charge species in the polymer electrolyte systems and also a constant phase element (CPE),which can be observed in the inserts of Figure 2. The CPEis shown at the region of low frequencies where the electrochemical doublelayer capacitance between the electrodes and the electrolytes was created. *Z_CPE_* impedance can be demonstrated as [44,45]:

(4) $$Z_{CPE}=\frac{1}{C\omega^{p}}\left[\cos\left(\frac{\pi p}{2}\right)-i\sin\left(\frac{\pi p}{2}\right)\right].$$

The parts of real (*Z_r_*) and imaginary (*Z_i_*) of the complex impedance (Z*) that are related to the EC (Figure 2a–f insert) are signified as follows:

(5) $$Z_{r}=R+\frac{\cos\left(\frac{\pi p}{2}\right)}{C\omega^{p}};$$

(6) $$Z_{i}=\frac{\sin\left(\frac{\pi p}{2}\right)}{C\omega^{p}},$$

where *C* is the capacitance of the CPE at the electrode and electrolyte interfaces and *P* is the spike/tail divergence from the horizontal axis. The parameters of the fitting in the EEC are indicated in Table 1.

The impedance plots show a spike inclined at an angle less than 90°and at low frequency, which is due to the capacitive component in the electrolytes [46]. This inclination also illustrates the effect of the blocking electrodes and the polarization effect within the electrolytes [47,48]. This phenomenon occurs because of the growth of the electric doublelayer as well as the accumulation of free charge at the electrolyte and electrode surface interface [49]. Based on Figure 2, there was a decrease in the bulk resistance with increasing glycerol concentration and zinc complex. The ionic conductivity of the CS:NH_4_F:Gly electrolyte systems can be calculated using the following equation [50,51]:

(7) $$\sigma_{dc}=\left(\frac{1}{R_{b}}\right)\times \left(\frac{t}{A}\right),$$

where *t* is the thickness of the sample while *A* represents the contact area of electrode–electrolyte. *R_b_* is the bulk resistance of the material that is taken from the interception of the spike with real axis (horizontal axis).

It is essential to determine the DC conductivity of the samples from the *R_b_* values using the above equation [52]. The DC conductivities were calculated and tabulated as shown in Table 2. It can be observed that the highest ionic conductivity of 1.71 × 10^−3^ S/cm was recorded by the plasticized electrolyte with the zinc complex (CSNHG5) with the lowest *R_b_* value. This proves that the metal complex inclusion into the plasticized system improved the electrolyte ionic conductivity. As documented by Amran et al. [53], the ionic conductivity of the electrolyte system of potato starch is chitosan (LiCF_3_SO_4_) and has been developed using glycerol inclusion from 7.65 × 10^−5^ S·cm^−1^ to 1.32 × 10^−3^ S·cm^−1^.The current work demonstrates that plasticizer and metal complexes are crucial to enhance DC conductivity. This might be linked to the improvement of the structure of amorphous. XRD results demonstrated in the above section also provide extra description.

Consequently, it is important for the polymer electrolytes to obtain a relatively high DC conductivity between 10^−5^ and 10^−3^ S/cm, which gives a promising performance for the application of the electrical doublelayer capacitors [54,55]. Therefore, it is proven that the glycerol and zinc complex increase the DC conductivity in the polymer electrolytes. The CSNHG5 electrolyte was chosen for the study of influence of zinc in the polymer complexes which can be seen in the next section.

### 3.3. EDLC Characteristics

#### 3.3.1. TNM Analysis

**Figure 3:**
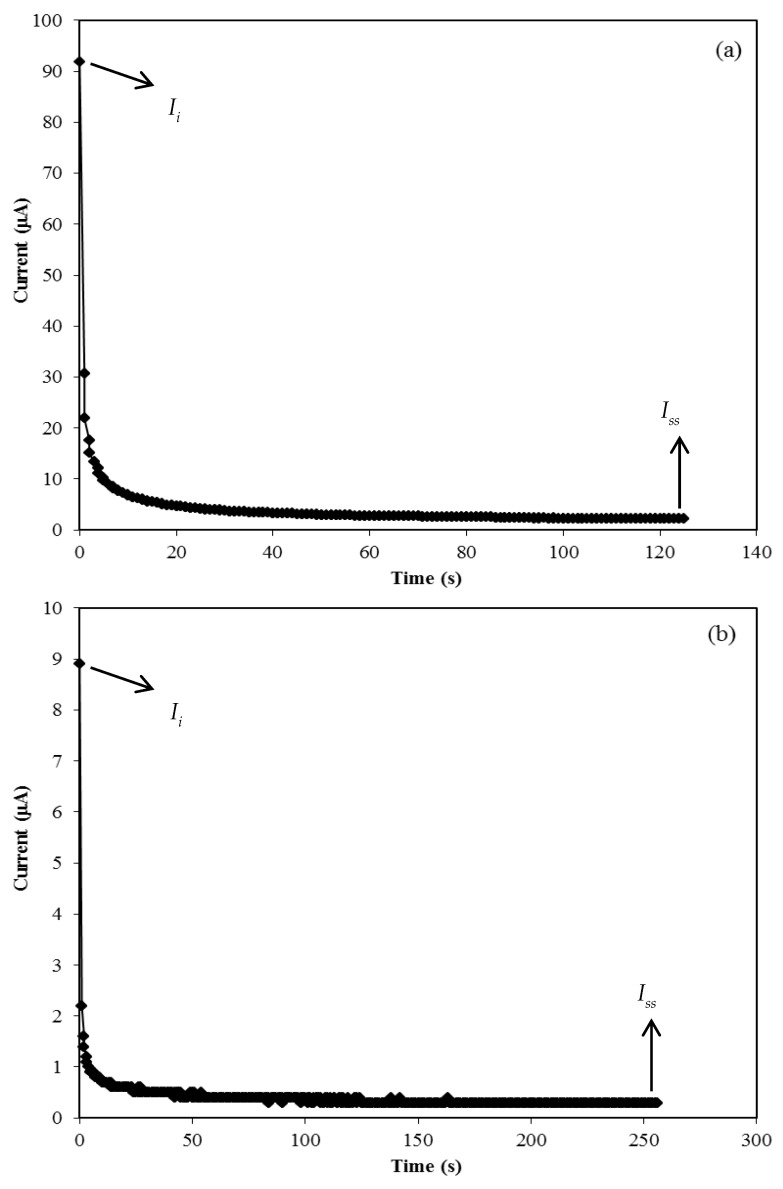
Current versus time for the (**a**) CSNHG4 electrolyte and (**b**) CSNHG5 electrolyte.

The transference number measurement (TNM) was carried out to determine the dominant charge carrier species within the electrolytes. Shukur et al. [56] reported that the polymer electrolyte system is dominated by ions if the transference number of ions (*t_ion_*) is near unity. The transference number of the ions and electrons (*t_e_*) is calculated using the following equations [57,58]:

(8) $$t_{ion}=\frac{I_{i}-I_{ss}}{I_{i}};$$

(9) $$t_{el}=1-t_{ion},$$

where *I_i_* and *I_ss_* represent the current at initial and steady state, respectively. Figure 3 shows a plot of the current against time during polarization for the CSNHG4 and CSNHG5 electrolytes.

From Figure 3, it can be seen that the initial current decreased with time for both systems. This was due to the depletion of ionic species within the electrolytes whichreached steady state when the movement of mobile ions was balanced by the diffusion process [29,59]. The current flow of ions is blocked by the stainless steel electrodes during the polarization process and only electrons can pass through [60]. The calculated values *t_e_* and *t_ion_* for both electrolyte systems are listed in Table 3. The value of *t_ion_* slightly decreased as the zinc was added into the glycerolized electrolyte. However, both systems show that ions are the dominant conducting species in the electrolytes and have been widely used in the application of electrochemical devices. This result is in good accordance with the glycerolized chitosan-NH_4_Br system as reported by Shukur et al. [31].

#### 3.3.2. LSV Study

**Figure 4:**
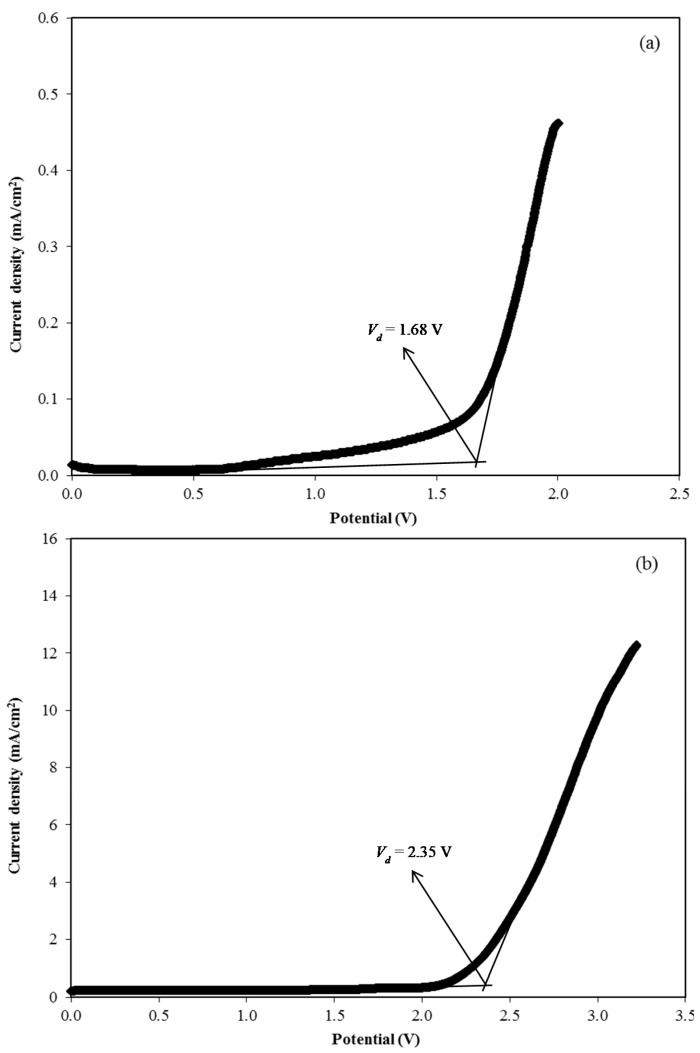
Linear sweep voltammetry (LSV) curve for the (**a**) CSNHG4 electrolyte and (**b**) CSNHG5 electrolyte.

The maximum operating voltage of an electrolyte at room temperature can be determined from the LSV study [61]. Figure 4 shows the LSV plot for the CSNHG4 and CSNHG5 electrolytes at 10 mV/s. It can be observed that there was no current flow when the voltage was below 1.68 V in the CSNHG4 electrolyte which means that no electrochemical reaction occurred [62]. The decomposition of the CSNHG4 electrolyte occurred at the voltage of 1.68 V. However, the value was found to increase with the addition of the zinc complex in the system up to 2.35 V, which is comparable with the value reported by Aziz et al. [52] for the chitosan/PEO–LiClO_4_ polymer electrolyte system. This shows that the potential window of the glycerolized electrolyte can be enhanced with the addition of the zinc complex. However, both electrolytes in this present work obtain a suitable decomposition voltage that is high enough to be applied in EDLCs that normally operate at 1.0 V [63].

#### 3.3.3. CV and EDLC Characterization

**Figure 5:**
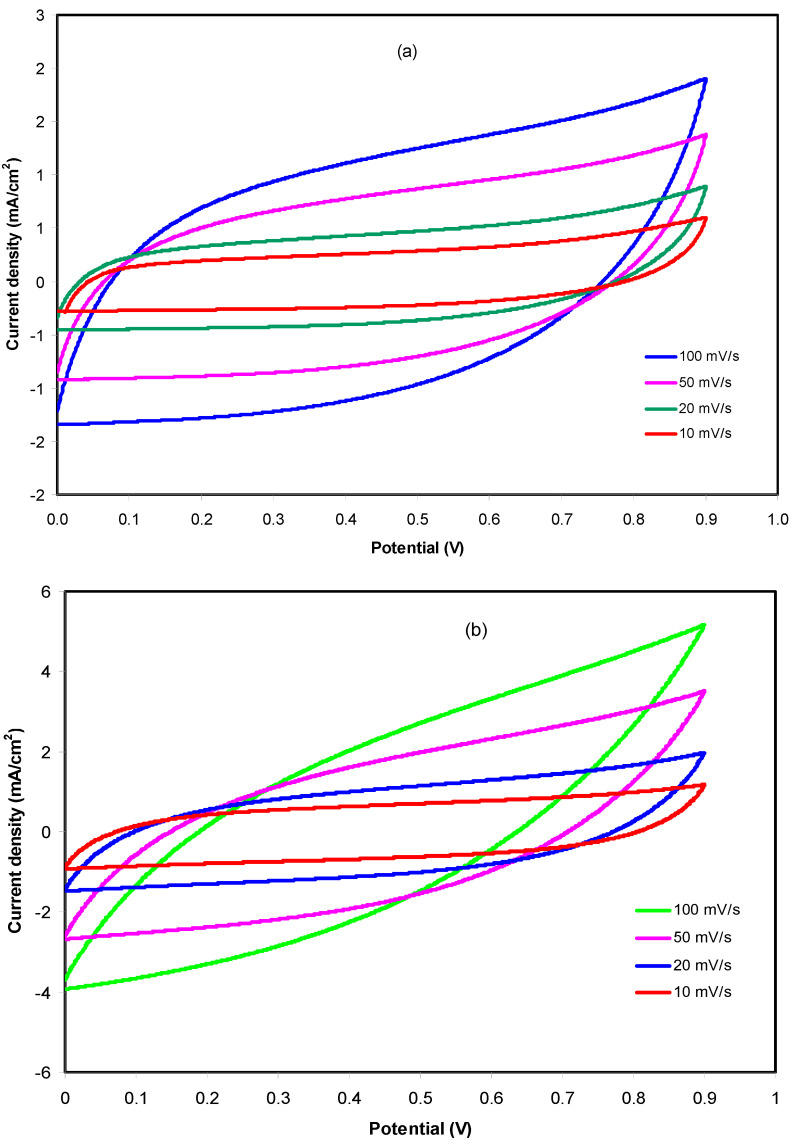
CV curves of the fabricated EDLC using (**a**) CSNHG4 and (**b**) CSNHG5 electrolyte at different scan rates.

CV analysis was conducted to test the charge storage behavior at the electrodes–electrolytes interface of an EDLC. The CV is carried out at different scan rates for both electrolyte systems and presented in Figure 5a,b.

Based on Figure 5a, it was observed that the curves turned from a leafshape to a rectangular shape as the scan rate decreased. The internal resistance and porosity of the carbon electrodes are the factors that cause the shape of the CV curves to become an imperfect rectangular shape [64]. There were no redox peaks observed in the CV curves of the fabricated EDLC using CSNHG4 electrolyte which indicates that neither oxidation nor reduction happens in the EDLC. The cations and anions in the EDLC will migrate to negative and positive electrodes, respectively, during the charging process. At the positive electrode, the induced electric field will attract anions and repel cations, while the opposite situation occurs at the negative electrode. The intense electric field holds the ions and electrons from electrolyte and electrode, respectively [65]. This phenomenon explains that a doublelayer charge is developed on the surface of carbon electrodes where the stored energy is the potential energy [66]. A similar pattern of curves was observed for the fabricated EDLC using the CSNHG5 electrolyte as shown in Figure 5b. The values of *C_s_* for both electrolyte systems at different scan rates werecalculated by using Equation (1) and tabulated in Table 4.

Generally, the *C_s_* values decreased when the scan rates increased. This is because the number of stored charges on the surface of the electrodes is reduced at higher scan rates, resulting in an increment of energy losses, hence the decrease in *C_s_* values [67]. However, the fabricated EDLC using the CSNHG5 electrolyte achieved higher *C_s_* values compared to the CSNHG4 electrolyte at every scan rate. This shows that the presence of the zinc complex in the glycerolized electrolyte helpsto improve the specific capacitance of the EDLC. In our earlier study, it was demonstrated that metalcomplexes expanded the structure of amorphous [32] and therefore more pathways wereavailable for ion conduction. As stated by a previous report [68], the enhancement in amorphous structure is helpful in moving local chain segments since it enhances ion movement and improves the DC ionic conductivity. Thus, ions freely move inside the polymer electrolyte. Rapid ion transportation in the polymer electrolyte also improves the adsorption of ions at the electrode–polymer–electrolyte interfaces, which offers larger *C_s_* of EDLC [69]. Therefore, the CSNHG5 electrolyte is chosen to be further analyzed in the EDLC fabrication studies.

**Figure 6:**
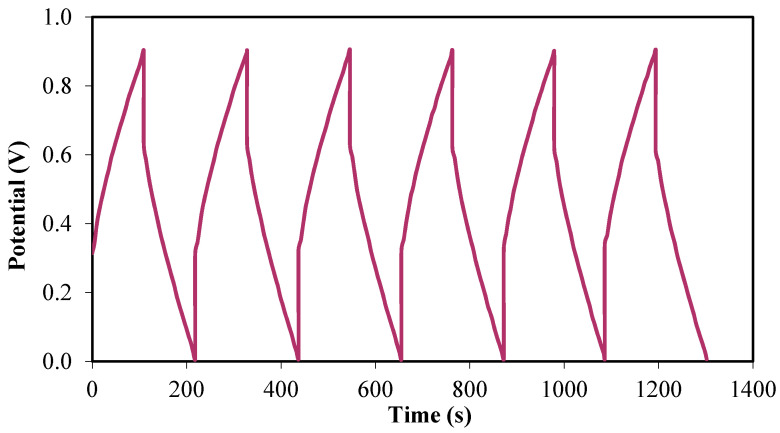
Charge–discharge profile for the fabricated EDLC at initial cycles.

The charge–discharge profile of the fabricated EDLC can further explain the cyclic durability of the EDLC as well as the charging and discharging processes that occurred within the system [70]. Figure 6 exhibits the charge–discharge curves of the EDLC at the initial cycles.

By using the galvanostatic charge–discharge profile, the capacitive behavior in the EDLC can be verified via the discharge slope that is almost linear [54]. The *C_s_* of the EDLC from the charge–discharge curves can be calculated by using Equation (2).

**Figure 7:**
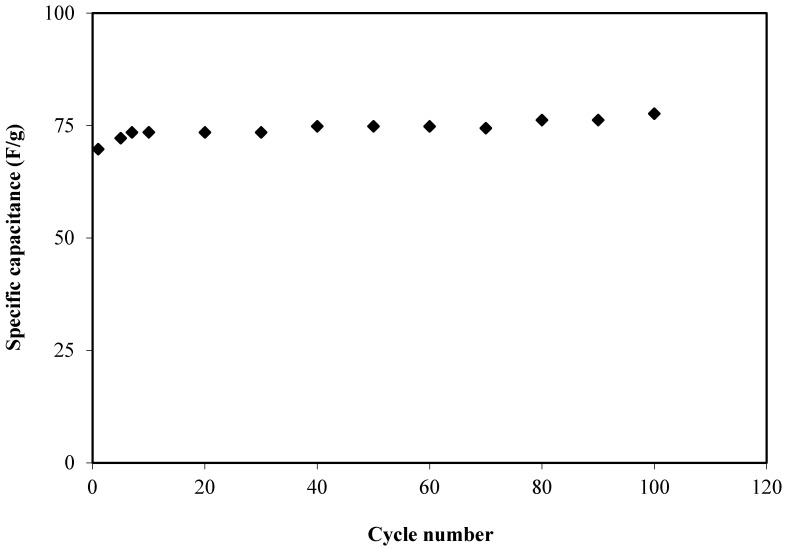
Specific capacitance (*C_s_*) of the fabricated EDLC for 100 cycles.

The calculated *C_s_* value of the EDLC for 100 cycles is presented in Figure 7. The *C_s_* at the firstcycle was found to be 69.7 F/g which was comparable to the *C_s_* value obtained from CV analysis which proves that EDLC exhibited the characteristics of a capacitor cell [71]. The *C_s_* increased to 73.5 F/g at the twentieth cycle and continued to increase to be almost constant at an average value of 75.6 F/g up to the one hundredthcycle. The *C_s_* achieved in this work is comparable to the *C_s_* value reported by Aziz et al. [54] for the fabricated EDLC which was at approximately 76.7 F/g for 100 cycles. Thus, the electrolyte studied in this current work can be established as a new material in the fabrication of EDLC with high specific capacitance.

**Figure 8:**
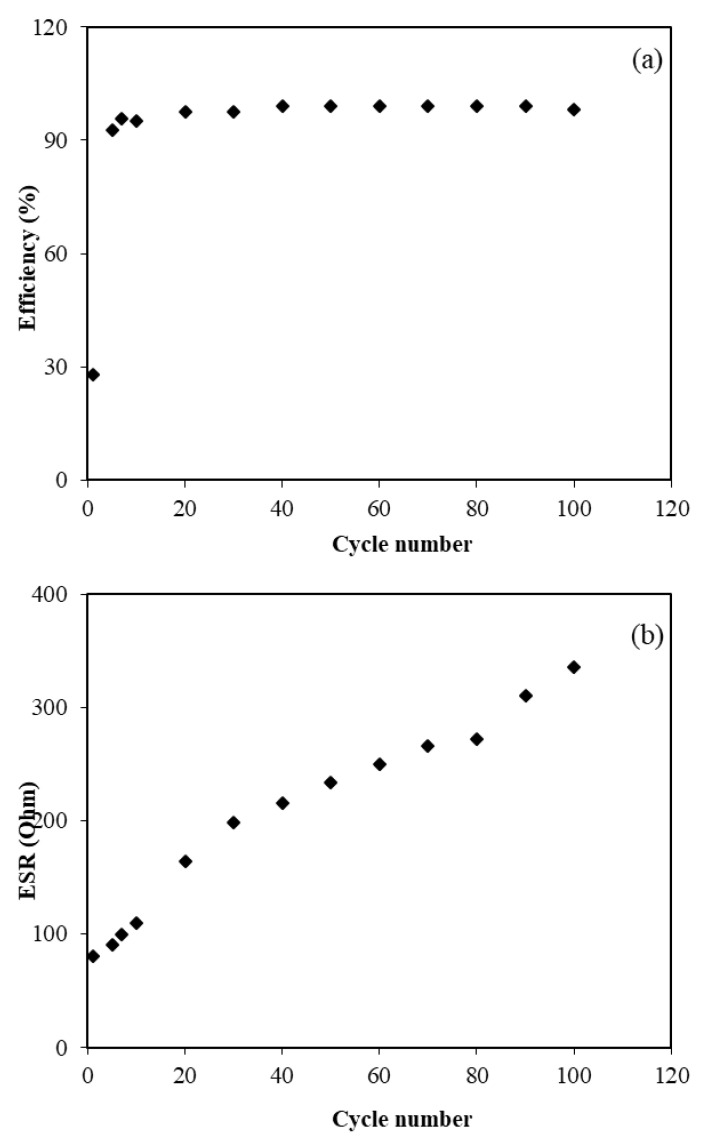
The plot of (**a**) efficiency, and (**b**) ESR of the fabricated EDLC for 100 cycles.

Coulombic efficiency (*η*) is a parameter that is used to study the cycling stability of the EDLC which can be calculated using the following equation [34]:

(10) $$\eta =\frac{t_{d}}{t_{c}}\times 100,$$

where *t_d_* and *t_c_* are the discharge and charge time, respectively. Figure 8a shows the efficiency of the fabricated EDLC for 100 cycles. The efficiency at the firstcycle was found to be 28.0% and then significantly increased to 95.1% at the tenthcycle. At the thirtiethcycle, efficiency was observed to be 97.5% and then remained constant at approximately 99.0% up to 100 cycles. It is thoughtthat the fabricated EDLC possesses plausible electrode–electrolyte contact as the efficiency was beyond 90.0% [72].

From the charge–discharge profile in Figure 6, it can be seen that there were tiny potential drops (*V_drop_*) before the discharging process began. This can be related to the existence of internal resistance in the EDLC, which is called equivalent series resistance (*ESR*). This *ESR* of the EDLC can be obtained from Equation (3). Figure 8b exhibits the ESRof the EDLC for the 100 cycles. It can be determined that the ESR value at the firstcycle was found to be 80 Ω and then increased to 234 Ω at the fiftieth cycle. It is notable that the value slightly increased up to 335 Ω throughout the 100 cycles.

As mentioned by Arof et al. [73], the internal resistance exists due to the charge and discharge process of electrolytes, type of current collectors (aluminum foils) and also the gap between the electrolyte and the electrode. The small value of ESR represents good contact between electrode and electrolyte and indicates that it is easy for ions to migrate toward the surface of the electrodes to form an electrical doublelayer [74]. A similar trend was reported for the chitosan/methylcellulose-NH_4_SCN system where the ESRvalues slightly increased while the *C_s_* values remained constant [54]. The rapid charging and discharging process will lead to the recombination of free ions and then the ion pair will be developed which leads to conductivity decrement.

Energy density (*E*) and power density (*P*) are also important to describe the performance of the EDLC. These parameters can be expressed by using the following equations [34,57]:

(11) $$E=\frac{C_{s}V^{2}}{2};$$

(12) $$P=\frac{V^{2}}{4m(ESR)}.$$

**Figure 9:**
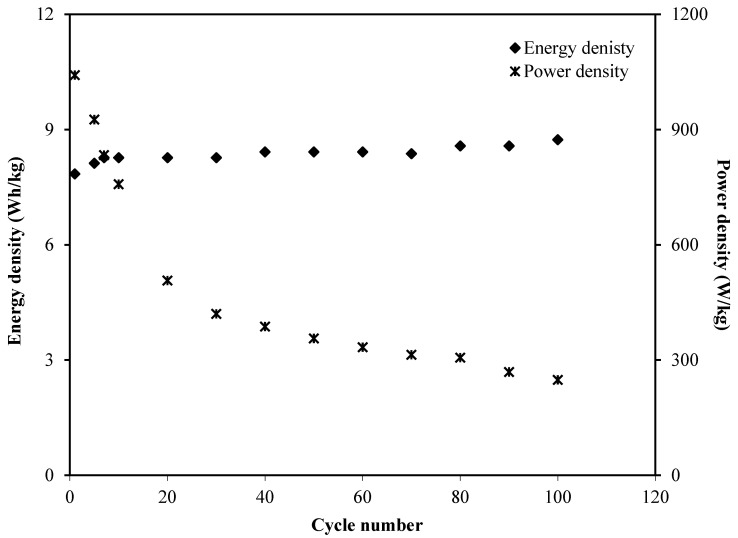
Energy density and power density of the fabricated EDLC for 100 cycles.

Figure 9 exhibits the calculated energy density as well as power density for the 100 cycles. The EDLC obtained the energy density of 7.8 Wh/kg at the first cycle and the value gradually increased to 8.3 Wh/kg at the tenth cycle and then remained stable around 8.5 Wh/kg from the thirtieth cycle towards the final cycle. This trend was harmonized with the pattern of *C_s_* illustrated in Figure 7. This result explains that the charge carriers require almost the same amount of energy to migrate towards the surface of the electrodes for the entire process of charge and discharge [75]. Moreover, the power density values werecalculated by using Equation (12), as plotted in Figure 9 where the *P* value at the firstcycle was found to be 1041.7 W/kg. The value then dropped to 506.9 W/kg at the twentiethcycle and slightly decreased to 248.2 W/kg until the EDLC completed 100 cycles. The trend of *P* is in agreement with the trend of theESR plot. This is because the depletion of the electrolyte occurred when the internal resistance increased which caused the recombination of ions due to the fast charging and discharging mechanism, thus reducing *P* at a high cycle number [76]. Both *E* and *P* values are clearly dependent on the mass loading of active material in the fabrication of EDLC.

## 4. Conclusions

The synthesis of glycerolized chitosan–NH_4_F solid polymer electrolyte has been successfully prepared using a solution cast technique. The CSNHG0 has the lowest ionic conductivity of 1.09 × 10^−4^ S/cm. With the addition of 40 wt.% glycerol, the ionic conductivity increased to 1.71 × 10^−3^ S/cm. The effect of zinc complex addition into the highest conducting electrolyte has been studied. The TNM measurements exhibited the transference number of ion (*t_ion_*) and electron (*t_e_*) for both systems. It showed that ions have been confirmed to be the dominant charge carrier in both electrolyte systems as *t_ion_* values for CSNHG4 and CSNHG5 electrolytes were 0.976 and 0.966, respectively. Based onthe LSV analysis, the zinc complex improved the electrochemical stability of CSNHG5 electrolyte to 2.25 V. The specific capacitance obtained by using the CV plot of the EDLC with the CSNHG5 electrolyte wasslightly higher compared to EDLC with theCSNHG4 electrolyte. The fabricated EDLC has been tested using CSNHG5 electrolyte for 100 cycles. The specific capacitance from the charge–discharge analysis ranged between 69.7 to 77.6 F/g over the 100 cycles. The equivalent series resistance increased from 80.0 to 335.7 Ω throughout the cycles. Finally, energy and power densities stabilized at the range of 7.8 to 8.5Wh/kg and 1041.7 to 248.2 W/kg, respectively, as the EDLC completed the cycles.

## Figures and Tables

**Table 1:** The EEC fitting parameters for all electrolyte systems at ambient temperature.

Sample	*P* (rad)	*K* (F^−1^)	*C* (F)
CSNHG0	0.68746	318,000	3.14 × 10^−6^
CSNHG1	0.738383	298,000	3.36 × 10^−6^
CSNHG2	0.738383	238,000	4.20 × 10^−6^
CSNHG3	0.776575	88,000	1.14 × 10^−5^
CSNHG4	0.814768	40,000	2.50 × 10^−5^
CSNHG5	0.814768	27,000	3.70 × 10^−5^

**Table 2:** Calculated DC conductivity for CS:NH_4_F:Gly electrolyte films at room temperature.

Sample Code	DC Conductivity (S/cm)
CSNHG0	(1.09 × 10^−4^) ± 0.19
CSNHG1	(1.26 × 10^−4^) ± 0.56
CSNHG2	(1.56 × 10^−4^) ± 0.47
CSNHG3	(6.59 × 10^−4^) ± 0.84
CSNHG4	(9.52 × 10^−4^) ± 0.93
CSNHG5	(1.71 × 10^−3^) ± 0.32

**Table 3:** Calculated transference numbers of ions and electrons.

Electrolyte	*t_ion_*	*t_e_*
CSNHG4	0.976	0.024
CSNHG5	0.966	0.034

**Table 4:** Specific capacitance (*C_s_*) of the fabricated EDLC using different electrolytes at various scan rates.

Scan Rate (mV/s)	Specific Capacitance, *C_s_* (F/g)	
	CSNHG4	CSNHG5
10	28.35	46.18
20	23.09	35.32
50	16.37	21.54
100	11.36	12.60
